# Burnout in nursing: causal factors and impacts on health and professional performance - a systematic review

**DOI:** 10.47626/1679-4435-2025-1432

**Published:** 2025-10-06

**Authors:** Thainara Caproni Batista, Isabella Nappi, Maria Victoria Moncada Xavier, Isabella Oliveira Fiuza, Camilla Briza Marques de Vasconcelos, Ricardo Toshio Enohi

**Affiliations:** 1 Universidade de Ribeirão Preto, Guarujá, SP, Brazil

**Keywords:** burnout, psychological burnout, nursing., esgotamento profissional, esgotamento psicológico, enfermagem.

## Abstract

Burnout syndrome is characterized as a state of physical and psychological exhaustion related to the work environment. The aim of this study is to analyze the main causal factors of burnout in nursing professionals and their impact on physical and mental health, as well as on professional performance. This is a systematic review that used databases such as SciELO and PubMed to search for articles in English and Portuguese from 2020 to 2025. The selection of articles, using the Preferred Reporting Items for Systematic Reviews and Meta-Analyses (PRISMA) protocol, was based on the following criteria: contributing factors to burnout, consequences of burnout, and prevalence of burnout. The selected articles were analyzed by two different reviewers, discarding those that did not fit the criteria described. It was concluded that overload, area of activity, and inadequate working conditions have a direct impact on this syndrome, resulting in a drop in professional performance, a decline in quality of life, and an impact on patient care.

## Abstract (pt)

A síndrome de *burnout* é caracterizada como um estado de esgotamento físico e psicológico relacionado ao ambiente de trabalho. O objetivo deste estudo é analisar os principais fatores causais do *burnout* em profissionais de enfermagem e seus impactos na saúde física e mental, bem como no desempenho profissional. Trata-se de uma revisão sistemática que utilizou bases de dados como SciELO e PubMed para buscar artigos em inglês e português, dentro de um período de 2020 a 2025. A seleção dos artigos, por meio do protocolo Preferred Reporting Items for Systematic Reviews and Meta-Analyses (PRISMA), baseou-se nos seguintes critérios: fatores contribuintes para o *burnout*, consequências do *burnout* e prevalência do *burnout*. Os artigos selecionados foram analisados por dois pareceristas distintos, descartando aqueles que não se enquadrassem nos critérios descritos. Concluiu-se que sobrecarga, área atuante e condições de trabalho inadequadas impactam diretamente nessa síndrome, resultando na queda do rendimento profissional, declínio na qualidade de vida e impacto no atendimento ao paciente.

## INTRODUCTION

Burnout syndrome is defined as a state of mental exhaustion related to the work environment, which leads to physiological changes. Among its most common symptoms are physical, psychological, and emotional exhaustion caused by work-related stress and overload. A total of 3 interdependent macro factors are required to characterize burnout syndrome: emotional exhaustion, depersonalization, and reduced personal accomplishment.^1^

Characterized by depersonalization and decreased professional fulfillment, burnout syndrome has become a major cause of psychological illness among nursing workers, with serious consequences for service quality and patient safety.^2^

In this context, one of the most affected groups is health professionals, who face a high risk of exhaustion in the workplace. Among health care fields, nursing stands out due to the constant pressure involved in its tasks and the diminishing ability to carry them out effectively. This issue becomes evident when such distress interferes with the efficiency of patient care, leading to reduced work quality, procedural errors, absenteeism, disengagement, dissatisfaction, conflicts with colleagues and family members, use of legal and illegal drugs, and physical inactivity.^3^

In 2020, 14.3% of nurses in Brazil were diagnosed with burnout syndrome, exhibiting physical and emotional symptoms such as irritability, headaches, shortness of breath, insomnia, anxiety, and a lack of interest in social interactions. In the workplace, this often led to reduced work quality, procedural errors, absenteeism, disengagement, dissatisfaction, interpersonal conflicts, substance use, and sedentary behavior.^4,5^

In Brazil, scientific studies on this topic remain scarce, even though it is a highly prevalent condition among the population today. Therefore, addressing burnout syndrome is essential to developing strategies that promote physical and mental well-being among nursing professionals in their work environment.^6^

The aim of this study is to analyze the main causal factors of burnout in nursing professionals and its impacts on physical and mental health as well as on professional performance.

## METHODS

A systematic review was conducted focusing on studies that address the impacts of burnout on the health of nursing professionals. The selection criteria included contributing factors to burnout, its consequences, and its prevalence. Databases used for the literature search were the Scientific Electronic Library Online (SciELO) and PubMed, covering the period from 2020 to 2025 and including articles in both Portuguese and English to broaden the scope of the review. Keywords used were “burnout,” “psychological exhaustion,” “occupational burnout,” and “nursing.” The search retrieved 5,505 articles from SciELO and 47,668 from PubMed.

Following the initial search, the Preferred Reporting Items for Systematic Reviews and Meta-Analyses (PRISMA) methodology was applied. The search results underwent a screening process, during which articles meeting the inclusion criteria were selected - totaling 16 - and submitted to 2 external reviewers. These evaluators assessed each study’s compliance with the established inclusion criteria.

**Figure 1:**
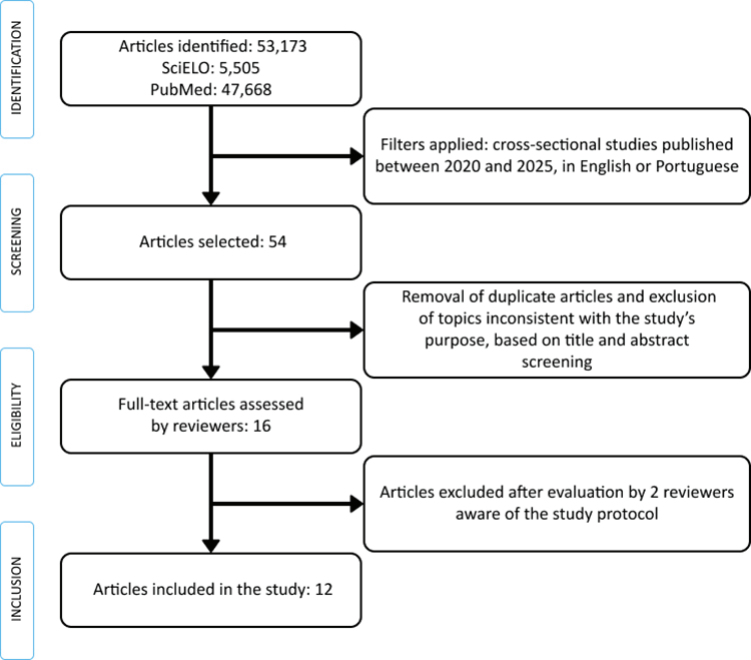
Preferred Reporting Items for Systematic Reviews and Meta-Analyses (PRISMA) flowchart for identification and screening of studies. SciELO = Scientific Electronic Library Online.

Of the 16 articles reviewed, 12 met the predefined criteria (Figure 1). Relevant data were extracted from these studies, including information on authors, year of publication, type of research, study period, objective, sample, location, results, and conclusions.

## RESULTS

The findings indicate that burnout syndrome negatively affects workers’ quality of life and well-being, both in the workplace and in their personal lives. Factors such as the nature of the work, lack of recognition, moral distress, and professional exhaustion were identified as key contributors to this condition.

Table 1 presents a summary of the articles selected for the systematic review, including author, year of publication, study type, location and study period, selected sample, main objective, and findings.

**Table 1:** Summary of articles selected for the systematic review

Author	Study type	Location/study period	Population or sample	Main objective	Findings
Villagran et al.^7^	Descriptive-analytical	Rio Grande do Sul/April to June 2019	Nurses working in a university hospital (n = 269)	To analyze the association between moral distress and burnout syndrome in nurses	An association between moral distress and burnout syndrome was observed, as well as among their dimensions
Medeiros et al.^8^	Cross-sectional	Pernambuco/June 2019 to February 2020	Nurses (n = 500): 110 from hospital A, 172 from hospital B, and 218 from hospital C	To identify possible associations between stress and distress and personal, work, and lifestyle variables	Professional exhaustion and lack of recognition are stressors that trigger defense mechanisms, including the desire to change jobs
Ribeiro et al.^4^	Cross-sectional	Campina Grande (Paraíba)/January to March 2018	Nursing professionals (n = 83) of both sexes and any age working in 2 emergency care units	To estimate the prevalence and associated factors of burnout syndrome and quality of life among nursing professionals	Burnout syndrome influences quality of life outcomes and is more prevalent among older professionals, those with higher incomes, and nurses
Möller et al.^9^	Cross-sectional, descriptive	Rio Grande do Sul/October 2018 to March 2019	Professionals from public (86 nursing technicians, 48 nurses) and private hospitals (80 nursing technicians, 21 nurses); n = 235	To evaluate and compare ICU nursing environments in public and private hospitals and the prevalence of burnout among nursing staff	Environmental control, autonomy, and support were considered critical points, highlighting the importance of institutional factors in improving working conditions
Almeida & Miclos^10^	Cross-sectional	São Paulo/October to November 2020	Professionals including 68 nurses and 127 nursing assistants; n = 207	To examine the association between authentic leadership and burnout syndrome among PHC nurses and analyze the relationship between positive psychological capital and burnout among subordinates	In nursing, burnout is associated with authentic leadership and psychological capital
Aragão et al.^11^	Cross-sectional	Bahia/July to November 2016	Nurses (n = 85)	To estimate the prevalence and associated factors of burnout syndrome among ICU nurses in a city in Bahia	The results may contribute to expanding discussions on stressful working conditions in ICUs
Soriano et al.^12^	Cross-sectional	Spain/September to November 2022	Surgical nurses (n = 214)	To estimate the prevalence of burnout among surgical nurses	Surgical nurses are at higher risk for burnout syndrome due to the nature of their work
Larysz et al.^13^	Cross-sectional	Poland/December 2019 to August 2020	Cardiac nurses (361 women and 39 men); n = 400	To determine the prevalence of depression and its association with burnout in cardiac nurses	Nurses showed high levels of depression and professional exhaustion, possibly negatively affecting patient care quality
Degree et al.^14^	Cross-sectional	Spain/August to October 2021	Nurse managers (n = 86) from various public hospitals in Spain	To analyze the prevalence and levels of burnout among nurse managers and identify its relationship with sociodemographic, occupational, and psychological factors	A total of 34.1% of participants reported high levels of burnout, expressed as low personal accomplishment; psychological and occupational factors play a significant role
De la Fuente-Solana et al.^15^	Multicenter, cross-sectional	Granada (Spain)/October 2019 to February 2020	Pediatric nurses (n = 95)	To demonstrate the impact of burnout among pediatric nurses and identify risk factors, providing insights for preventive health strategies	Neuroticism and depression were associated with increased vulnerability to burnout
Velando-Soriano et al.^16^	Cross-sectional	Spain/August to October 2021	Surgical nurses (n = 214) from 23 hospitals in Andalusia (Spain)	To determine burnout levels among surgical nurses in Andalusia (Spain)	Surgical nurses show high levels of burnout. Evidence links burnout to personality traits and sociodemographic variables
Zanatta & Lucca^17^	Descriptive with cross-sectional design and quantitative approach	São Paulo/March to September 2012	Health professionals (n = 188)	To identify the prevalence of burnout syndrome among physicians, nurses, and nursing technicians working in a pediatric onco-hematology hospital in São Paulo	Health professionals are highly vulnerable to burnout syndrome

ICU = intensive care unit; PHC = primary health care.

In order to analyze the association between moral distress and burnout syndrome in nurses, a group of 269 nurses working at a university hospital was evaluated. Among them, 30.9% reported high emotional exhaustion and exhibited moderate levels and frequency of moral distress, establishing a link between moral distress and work-related exhaustion.^7^

A cross-sectional study involving a sample of 500 nurses - 110 from hospital A, 172 from hospital B, and 218 from hospital C - was conducted to assess potential associations between occupational stress and personal, work-related, and lifestyle variables. The results confirmed that professional exhaustion was rated as “satisfactory” in 19.4% of the nurses, “critical” in 47.1%, and “severe” in 33.5%.^8^

Another study conducted in the state of Paraíba, Brazil, used a sample of 83 nursing professionals working in emergency care units to assess quality of life and determine the prevalence of burnout syndrome in this population. The findings revealed that 78.3% of participants had low professional efficacy, 53% had moderate depersonalization, and 55.4% had moderate emotional exhaustion.^4^

Regarding the prevalence of burnout among intensive care unit (ICU) nursing teams from both public and private hospitals, a study conducted in Porto Alegre, Rio Grande do Sul, indicated that the work environments were considered favorable for nursing practice. Burnout prevalence among nursing professionals ranged from 2.5% to 9.5%, classified as low in both hospitals. Additionally, moderate levels were observed in the subscales of professional accomplishment, emotional exhaustion, and depersonalization. In both hospitals, nursing teams reported high job satisfaction, a positive perception of care quality and safety, and low intention to leave their jobs.^9^

Concerning workplace hierarchy and leadership dynamics, an analysis carried out in primary health care units (UBSs) in São Paulo found that subordinates were more vulnerable than their supervisors to developing burnout syndrome. A total of 192 nursing professionals were surveyed, including 12 leaders and 180 subordinates. According to the leaders, no significant associations were found between burnout scales and authentic leadership. However, among subordinates, a significant negative association was observed between burnout scores and leadership dimensions.^10^

An analysis aimed at estimating the prevalence and associated factors of burnout syndrome among ICU nurses in a city in the state of Bahia showed that the overall exhaustion rate was 53.6%. This result was obtained through a cross-sectional study involving 65 ICU nurses, which revealed associations with age, tobacco use, alcohol consumption, weekly night shift hours, employment contract type, ICU specialization, number of patients per shift, monthly income, and perception of the job as active or highly demanding.^11^

In order to determine the levels of burnout among surgical nurses in Andalusia, Spain, the stage of burnout was identified for each participant, along with its relationship to sociodemographic, occupational, and personality-related variables. Key findings indicated that 29.4% of nurses exhibited high emotional exhaustion, 25.7% experienced depersonalization, and 28% had low levels of personal accomplishment.^12^

Using a sample of 400 cardiac nurses (361 women and 39 men) in Poland, the strongest correlation was found between depression and emotional exhaustion, followed by a slightly weaker correlation with depersonalization and the weakest with professional accomplishment.^13^

In order to analyze the prevalence and levels of burnout among nurse managers and examine its relationship with sociodemographic, occupational, and psychological factors, a study was conducted with 86 nurses specializing in administration and management. The study found that 34.1% of participants were classified as having a high level of burnout and that shift-working nurses were more likely to develop the syndrome than those not working shifts.^14^

A study carried out in Granada, Spain, surveyed 95 nurses working in 4 different hospitals. According to data, 22% of pediatric nurses showed high levels of emotional exhaustion, 18.5% high depersonalization, and 39.6% low personal accomplishment. In total, 38.6% of participants exhibited high levels of burnout, especially in terms of low personal accomplishment.^15^

A cross-sectional study conducted in Spain interviewed 214 surgical nurses from 23 hospitals in the Andalusia region to assess burnout levels. The results showed that 38.8% had low levels of burnout syndrome, 31.8% had moderate levels, and 29.4% had high levels, indicating a direct relationship between job demands and occupational exhaustion.^16^

An exploratory study in a pediatric onco-hematology hospital in São Paulo aimed to analyze the prevalence of burnout syndrome among physicians, nurses, and nursing technicians. According to the hospital’s employee records, it had 65 nurses, of whom 29.8% showed high depersonalization and low personal accomplishment, while 3.5% met criteria for all 3 core dimensions of burnout syndrome.^17^

## DISCUSSION

### CAUSAL FACTORS

Stress, work overload, emotional exhaustion, dissatisfaction, frustration, lack of appreciation, insufficient recognition of effort and performance, indignation, and a sense of injustice are recurring elements in the suffering reported by nurses. Low wages, combined with lack of recognition, represent one of the main sources of stress, often leading professionals to reconsider their careers.^8^

Although ICU hospital environments were evaluated positively in terms of being favorable to nursing practice, subscale analyses revealed that private hospitals scored below ideal levels regarding autonomy, environmental control, and organizational support. These findings highlight that nursing professionals continue to suffer due to inadequate working conditions, which include physical, mental, and emotional overload.^9^

Another relevant point is the heightened risk of burnout among surgical nurses, largely due to the nature of their work. More than half of the professionals in this field exhibited moderate to high levels of emotional exhaustion and depersonalization.^12^

### MOST VULNERABLE GROUPS

In terms of leadership, subordinates appear more susceptible to developing burnout syndrome, whereas nurse leaders did not show a significant association with the condition.^10^

Work-related exhaustion is also more prevalent among ICU nurses, likely due to the greater workload in these settings. The most affected individuals in this group are those aged 34 or younger, smokers, alcohol users, night shift workers (up to 24 hours), ICU specialists, and those with a monthly income of up to R$ 3,000.^11^

Among the different nursing specialties, surgical nurses represent a high-risk group for burnout syndrome, mainly due to the psychological and physical demands of the work environment. Key determining factors include marital status and gender, with married women being the most vulnerable.^12,16^ In this context, a study showed a higher prevalence among childless women with formal employment, who held more than 2 jobs and had worked for an average of 5 to 9 years.^17^

### IMPACT AND CONSEQUENCES

Of the 12 studies analyzed, four clearly demonstrated the impact of burnout on nurses’ work and personal lives, identifying factors such as moral distress, low professional efficacy, depersonalization, emotional exhaustion, feelings of low personal accomplishment, and negative effects on patient care. The findings of these studies point to significant harm to the mental health of affected nursing professionals.

Burnout syndrome also interferes with overall quality of life, with notable effects in the domains of vitality, pain, social functioning, and mental health. Additionally, the greater the severity of burnout, the lower the reported quality of life among participants.^4^

The most evident dimension of this scenario is low personal accomplishment. On-call duties and professional responsibility are associated with greater personal fulfillment, whereas psychological variables such as depression and neuroticism are the most significant predictors of burnout.^12^

Moreover, nurses experiencing high levels of depression and professional exhaustion may negatively affect the quality of patient care.^13^

## CONCLUSIONS

This article sought to investigate the causes, consequences, and impact of burnout on the daily lives of nurses. Most of affected professionals identified in the studies were nurses in their third decade of life, working night shifts of up to 24 hours, specialized in ICU or surgical care, and earning up to R$ 3,000.00 per month.

The reviewed studies demonstrate that inadequate working conditions for nurses - particularly physical, mental, and emotional overload - are major contributing factors to burnout.

The main consequences identified include moral distress, low professional efficacy, emotional exhaustion, and, most critically, compromised patient care.

Therefore, it is essential to investigate urgent interventions to mitigate the effects and manifestations of moral distress and burnout syndrome by developing strategies aimed at safeguarding workers’ health.
